# Knockdown of MTDH Sensitizes Endometrial Cancer Cells to Cell Death Induction by Death Receptor Ligand TRAIL and HDAC Inhibitor LBH589 Co-Treatment

**DOI:** 10.1371/journal.pone.0020920

**Published:** 2011-06-08

**Authors:** Xiangbing Meng, Pavla Brachova, Shujie Yang, Zhi Xiong, Yuping Zhang, Kristina W. Thiel, Kimberly K. Leslie

**Affiliations:** 1 Department of Obstetrics and Gynecology, The University of Iowa, Iowa City, Iowa, United States of America; 2 Holden Comprehensive Cancer Center, The University of Iowa, Iowa City, Iowa, United States of America; University of Illinois at Chicago, United States of America

## Abstract

Understanding the molecular underpinnings of chemoresistance is vital to design therapies to restore chemosensitivity. In particular, metadherin (MTDH) has been demonstrated to have a critical role in chemoresistance. Over-expression of MTDH correlates with poor clinical outcome in breast cancer, neuroblastoma, hepatocellular carcinoma and prostate cancer. MTDH is also highly expressed in advanced endometrial cancers, a disease for which new therapies are urgently needed. In this present study, we focused on the therapeutic benefit of MTDH depletion in endometrial cancer cells to restore sensitivity to cell death. Cells were treated with a combination of tumor necrosis factor-α-related apoptosis-inducing ligand (TRAIL), which promotes death of malignant cells of the human reproductive tract, and histone deacetylase (HDAC) inhibitors, which have been shown to increase the sensitivity of cancer cells to TRAIL-induced apoptosis. Our data indicate that depletion of MTDH in endometrial cancer cells resulted in sensitization of cells that were previously resistant in response to combinatorial treatment with TRAIL and the HDAC inhibitor LBH589. MTDH knockdown reduced the proportion of cells in S and increased cell arrest in G2/M in cells treated with LBH589 alone or LBH589 in combination with TRAIL, suggesting that MTDH functions at the cell cycle checkpoint to accomplish resistance. Using microarray technology, we identified 57 downstream target genes of MTDH, including calbindin 1 and galectin-1, which may contribute to MTDH-mediated therapeutic resistance. On the other hand, in MTDH depleted cells, inhibition of PDK1 and AKT phosphorylation along with increased Bim expression and XIAP degradation correlated with enhanced sensitivity to cell death in response to TRAIL and LBH589. These findings indicate that targeting or depleting MTDH is a potentially novel avenue for reversing therapeutic resistance in patients with endometrial cancer.

## Introduction

A common problem with cancer therapy is the development of resistance, and an improved understanding of the underlying pathways involved with drug resistance could lead to the development of new strategies to overcome this resistance. A recently discovered gene, metadherin (MTDH, also known as AEG-1 or LYRIC) has emerged as a potentially crucial mediator of tumor progression, metastasis, and resistance to chemotherapies [1], [2], [3], [4]. MTDH is proposed to promote tumor progression through the integration of multiple signaling pathways including ras, myc, Wnt, PI3K/AKT, and NF-κB in various types of cancer cells [4], [5], [6], [7], though the mechanism by which MTDH controls these signaling events is unclear. In this study, we investigated the role of MTDH in endometrial cancer and its inhibition as a mechanism to overcome drug resistance.

Initial interest in MTDH as a factor in chemoresistance arose as a consequence of NCI60 pharmacogenomic data, which found that the genomic copy number gain on 8q22 is a defining event in chemoresistance [8]. An earlier study reported that lymph node metastasis is significantly associated with copy number gains at 8q22–q23 in endometrial cancers [9]. Thus far, MTDH is the only known gene on 8q22 that has been shown to correlate with poor clinical outcomes in patients with solid tumors [8]. *In vitro* and *in vivo* chemoresistance analyses confirmed that MTDH knockdown sensitizes various types of tumors — including breast cancer, hepatocellular carcinoma, prostate cancer, and neuroblastoma— to multiple chemotherapy agents such as 5-fluorouracil (5-FU), cisplatin, paclitaxel, and doxorubicin [1], [10], [11]. However, the ability of MTDH knockdown to sensitize cells to targeted therapies, which have come to symbolize the future of cancer therapeutics, has not yet been explored.

Tumor necrosis factor (TNF)-α-related apoptosis-inducing ligand (TRAIL) recently emerged as a promising targeted therapeutic strategy in various types of cancers due to its pro-apoptotic characteristics [12], [13]. As a member of the TNF family, TRAIL specifically activates extrinsic apoptotic pathways in cancer cells by binding to death receptors. Importantly, TRAIL selectively promotes apoptosis of tumor cells but has no effect on normal human reproductive tract cells including those in the endometrium, ovary, cervix, or fallopian tube [13]. Some cancer cells are resistant to TRAIL-induced apoptosis [14], [15], [16], therefore combinatorial regimens have been adopted to restore sensitivity [13], [17]. In several studies, histone deacetylase (HDAC) inhibitors have been demonstrated to further increase sensitivity to TRAIL-induced apoptotic cell death [18], [19], [20], [21]. Unfortunately some cancer cells remain resistant to combined TRAIL and HDAC inhibitor treatment [22], and new approaches to restore sensitivity to these targeted therapies are necessary.

We examined the effect of depleting MTDH levels on restoring sensitivity to TRAIL-based targeted therapies. The data reported herein demonstrate that MTDH regulates cell cycle and cell survival in response to treatment with HDAC inhibitors and TRAIL, suggesting that MTDH is a promising therapeutic target to increase the efficacy of TRAIL and HDAC inhibitor combinatorial treatment.

## Results

### MTDH expression is elevated in endometrial cancer cell lines and tissues

**Figure 1:**
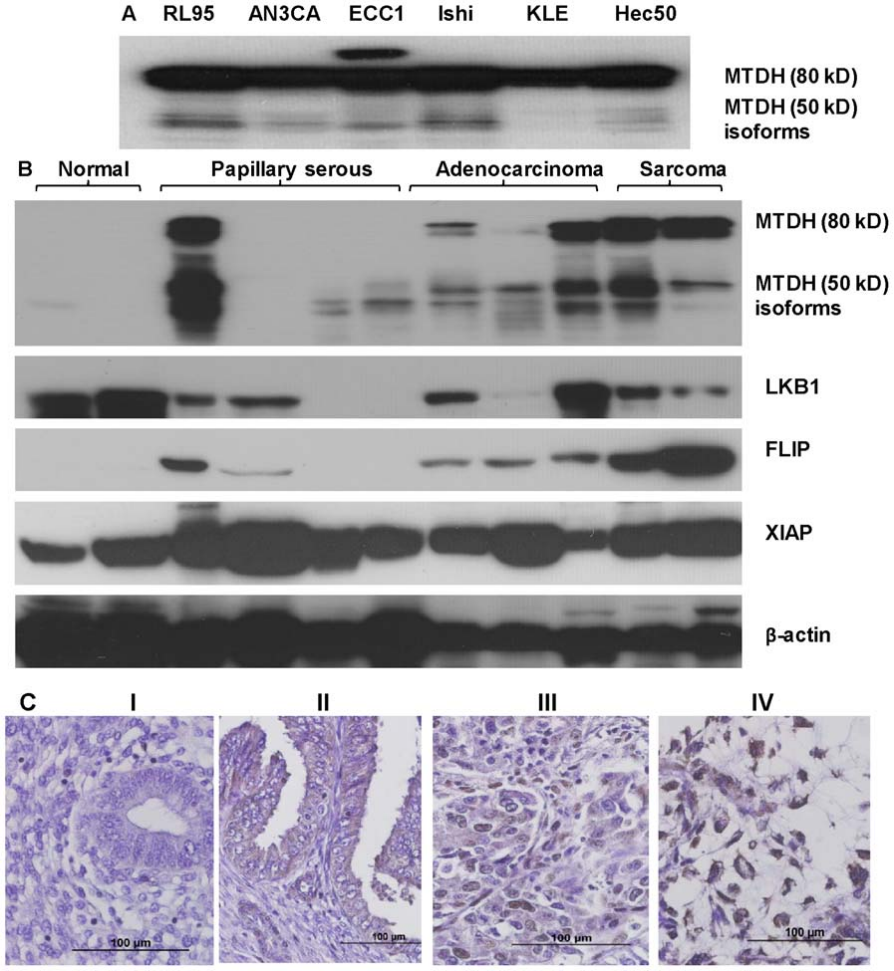
Overexpression of MTDH was detected in endometrial cancer cell lines and tissues. (A) Expression of MTDH was detected in all of the six tested endometrial cancer cell lines: RL95, AN3CA, ECC1, Ishikawa H (Ishi), KLE and Hec50 cells. (B) Expression of MTDH was detected in endometrial tissues from two normal individuals, four patients with papillary serous, three patients with adenocarcinoma, and two patients with endometrial sarcoma. Expression of the tumor suppressor gene LKB1 and two anti-apoptotic genes, XIAP and FLIP, was also detected by Western blotting in the same samples. (C) MTDH expression was detected by immunohistochemistry staining using the disease spectrum (endometrial cancer progression) tissue array with over 50 samples of endometrial tissues from benign to cancer. (i) normal endometrial tissue, (ii) low grade endometrial adenocarcinoma, (iii) high grade endometrial adenocarcinoma, and (iv) endometrial cancer metastatic to the abdominal cavity. Original magnification, ×400.

MTDH was highly expressed at the protein level in all six endometrial cancer cell lines tested (RL95, AN3CA, KLE, Ishikawa, Hec50co and ECC1, Figure 1A). In endometrial cancer patient tissues, MTDH expression was elevated compared to normal endometrium (Figure 1B). Specifically, the expression of 80 kDa MTDH and putative 50–55 kDa MTDH isoforms were significantly higher in endometrial cancer samples including papillary serous, sarcoma, and adenocarcinoma, whereas MTDH was undetectable in normal endometrial tissues (Figure 1B). Because no MTDH was detected in normal endometrial tissue, we blotted for the tumor suppressor LKB1 as a control (Figure 1B). Increased expression of cytoplasmic MTDH in endometrial adenocarcinoma and nuclear MTDH in some metastatic endometrial cancer was also observed in endometrial cancer tissues as shown in Figure 1C by immunohistochemistry with an MTDH-specific antibody. XIAP and FLIP are two pro-survival proteins associated with the death receptor-induced extrinsic apoptotic pathway [12]. We therefore examined whether there is a correlation between expression of pro-survival proteins XIAP and FLIP with MTDH expression. While expression of MTDH and XIAP did not correlate, we did detect a correlation with FLIP expression (Figure 1B). The increased expression of MTDH in all cancerous cells and tissues examined suggests that it may play a role in endometrial carcinogenesis.

### Knockdown of MTDH reduces colony formation

**Figure 2:**
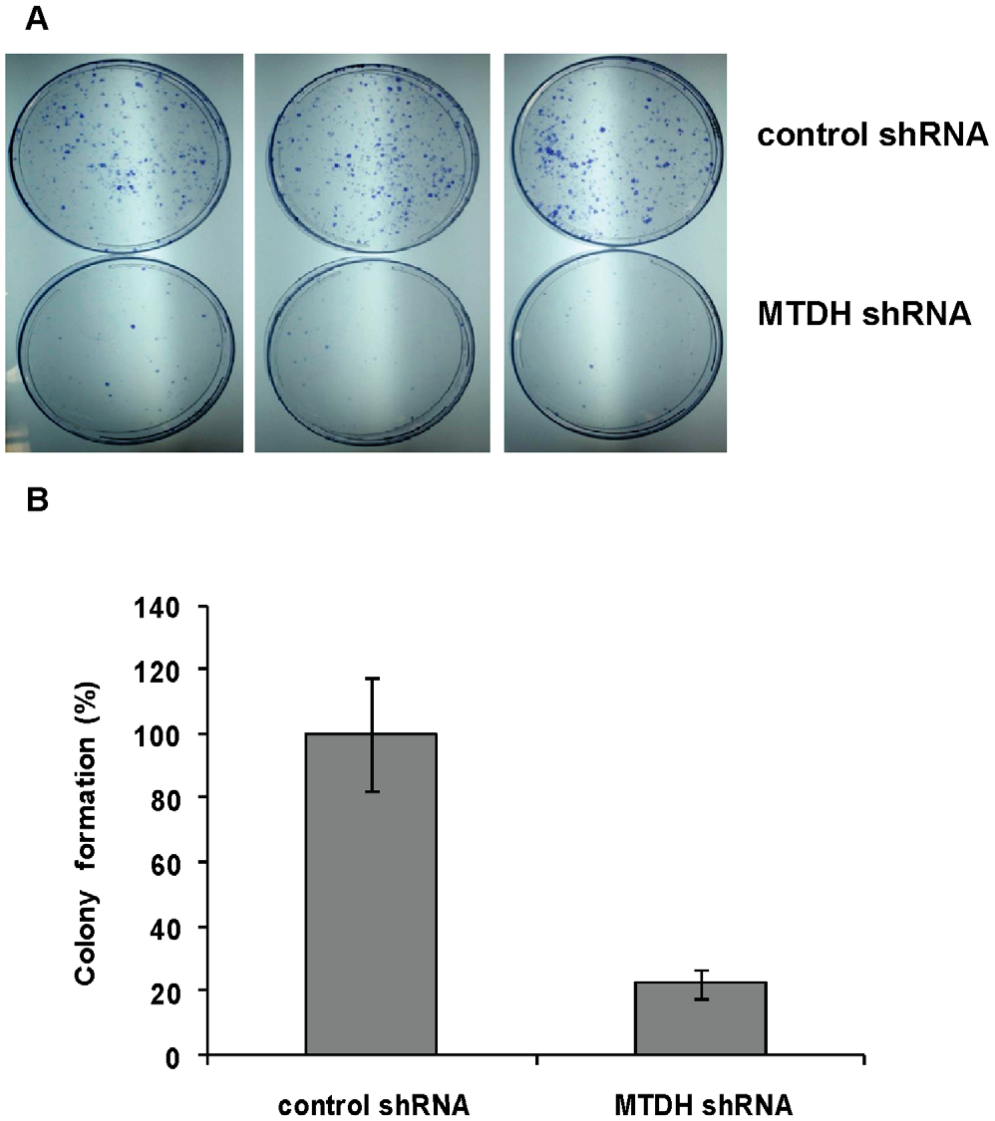
MTDH knockdown inhibits Hec50co colony formation. Hec50co cells were transfected with control or MTDH shRNA expression vectors. (A) Colonies formed by control or MTDH shRNA-transfected Hec50co cells are shown after the transfection of shRNAs and after selection with 2 ug/ml puromycin for 3 weeks. (B) Quantification of the relative colony formation in control versus MTDH knockdown Hec50co cells.

Due to the elevated expression of MTDH in the various endometrial cancer cell lines and human endometrial cancer samples, we next examined the tumorigenic potential of MTDH in the Type II endometrial cancer cell line Hec50co using a short-hairpin RNA (shRNA) specific for MTDH. A 3.49-fold reduction in MTDH gene expression was detected by quantitative real-time PCR (qRT-PCR) in MTDH shRNA-transfected Hec50co cells compared to control scrambled shRNA-transfected cells (Table S1). Depletion of MTDH decreased colony formation in cells stably expressing MTDH shRNA compared to control shRNA (Figure 2A, B). These results were confirmed using three shRNA constructs targeting different regions of MTDH. A similar phenotype was also observed upon knockdown of MTDH in Ishikawa H Type I endometrial cancer cells, indicating that elevated MTDH expression is generally required for endometrial cancer colony formation.

### Identification of MTDH-regulated genes in endometrial cancer cell lines

**Figure 3:**
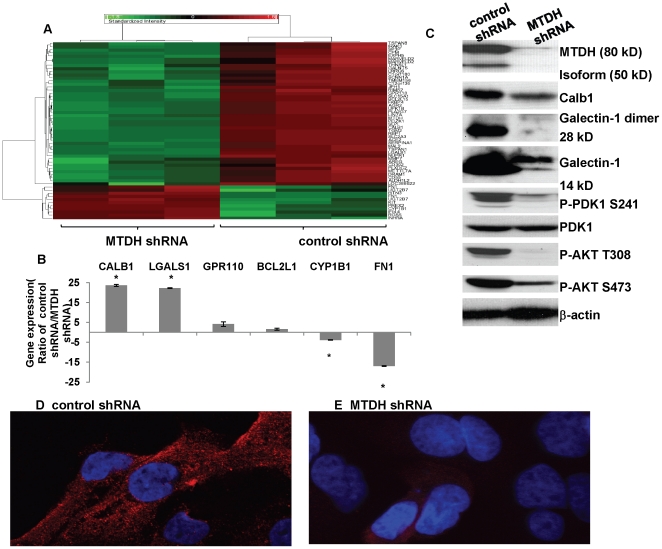
MTDH knockdown causes alterations in gene expression and PI3K/AKT signal transduction in Hec50co cells. (A) Heat map of two-fold change of gene expression (as determined from Affymetrix array data) between control and MTDH knockdown Hec50co cells. (B) Gene expression changes were validated by qRT-PCR for the indicated genes identified in the microarray (* p<0.05). (C) Expression and/or phosphorylation of the indicated proteins were examined in Hec50co cells stably expressing either control shRNA or MTDH shRNA. Total PDK1 and β-actin were used as loading controls. (D,E) Expression of PIP3 was detected in control (D) or MTDH knockdown (E) Hec50co cells by PIP3 immuno-fluorescent staining.

To identify downstream genes mediating the tumorigenic effects of MTDH in endometrial cancer cell lines, an Affymetrix oligonucleotide microarray (Human gene array 1.0 ST) was used to detect differentially expressed genes in Hec50co cells stably transfected with either MTDH shRNA or a control shRNA (Data have been deposited on GEO, GSE26134). Depletion of MTDH by knockdown resulted in at least two-fold gene expression alteration of 57 genes (Figure 3A). Several of these genes were randomly chosen to confirm the changes by qRT-PCR (Figure 3B).

Data from the array indicated that MTDH regulates expression of genes involved in invasion, tight junctions, as well as chemoresistance (Table S1 and S2). For example, galectin-1 (LGALS1) and calbindin 1 (Calb1) were markedly down-regulated at the mRNA (Figure 3B) and protein levels (Figure 3C) in MTDH shRNA-transfected Hec50co cells. Calb1 and galectin-1 are two putative MTDH downstream genes that may regulate the metabolism of phosphatidylinositol, which is part of the PI3K/AKT signaling pathway commonly upregulated in endometrial cancer [23]. We also examined the changes in gene expression in Ishikawa H endometrial cancer cells in response to MTDH silencing and observed some overlap with genes that were altered in Hec50co cells (Table S3 and S4).

We next sought to understand how MTDH regulates the PI3K/Akt pathway. Expression of galectin-1 is upregulated in various types of tumors and is reported to mediate tumor invasion and metastasis by increasing expression of matrix metalloproteinase MMP-9 and MMP-2 and promoting reorganization of the actin cytoskeleton [24]. Consistent with a previous study in which overexpression of MTDH promotes activation of the PI3K/AKT pathway in neuroblastoma [7], we detected an inhibition of components of PI3K/AKT signaling pathway in Hec50co cells with depleted MTDH as compared to control (Figure 3C). Specifically, a dramatic reduction in PDK1 phosphorylation at S241 and AKT phosphorylation at both T308 and S473 occurred in MTDH knockdown cells, although no significant change of total PDK1 protein level was detected (Figure 3C). Furthermore, as shown in Figure 3D and E, a dramatic reduction in the level of PIP3 was detected in Hec50co cells with MTDH shRNA as compared to cells with control shRNA. These findings demonstrate that MTDH can activate the pro-survival pathways of PI3K/AKT via up-regulation of PIP3.

### MTDH knockdown sensitizes cells to targeted cancer therapies

**Figure 4:**
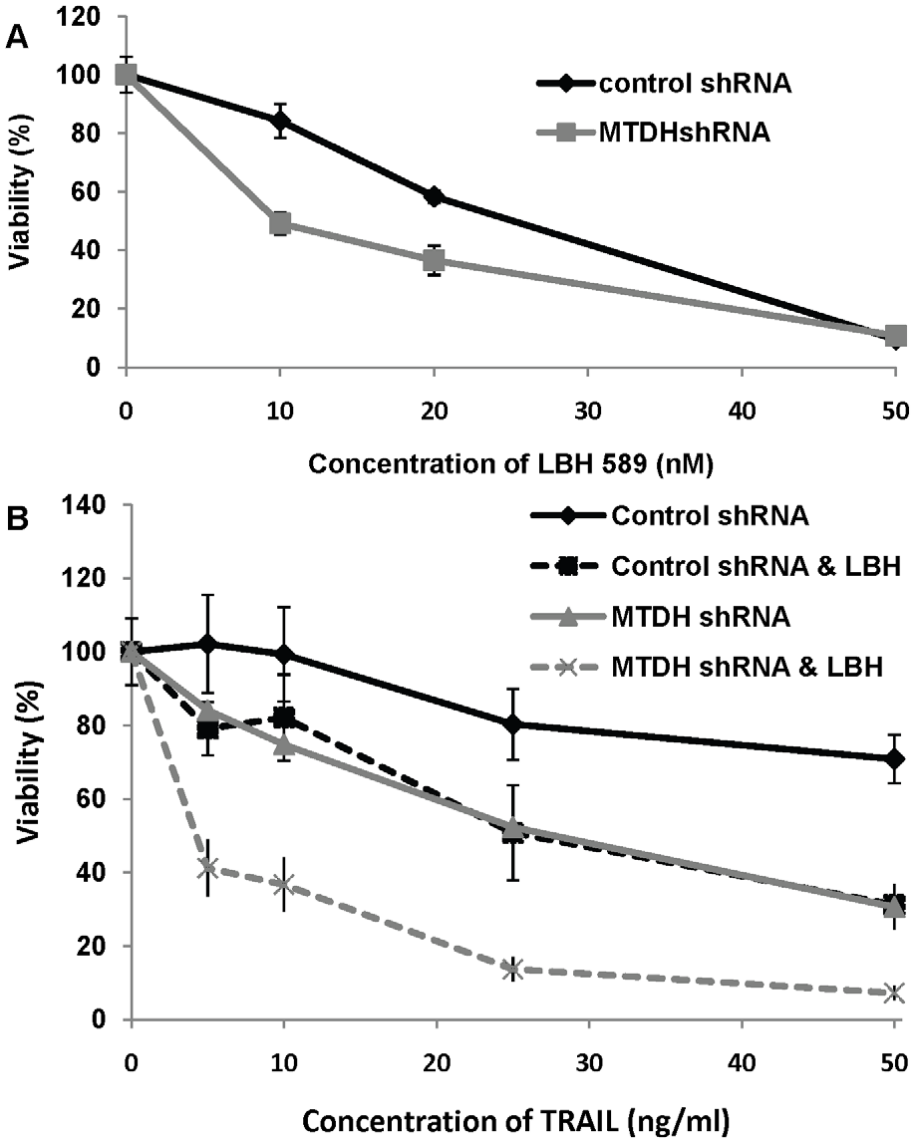
MTDH knockdown increases cell death induction by the HDAC inhibitor LBH589 alone or in combination with TRAIL. (A) Cell death induction by LBH589 as a single agent was detected in control or MTDH knockdown Hec50co cells. After 3 days, cell death was determined by the WST-1 method. (B) Cell death resulting from different concentrations of TRAIL (5 to 50 ng/ml) alone or in combination with 5 nM LBH589 was analyzed in control or MTDH knockdown Hec50co cells.

To overcome resistance to therapies, HDAC inhibitors such as LBH589 have been used to restore TRAIL-induced apoptosis in various types of cancers [18], [19]. Hec50co cells were previously shown to be resistant to TRAIL-mediated apoptosis [13]. Therefore, to test the influence of MTDH on sensitivity to TRAIL in these cells, we treated Hec50co cells with or without MTDH (control vs. MTDH knockdown) with combined TRAIL and LBH589 treatment for three days and then examined cell viability. Compared with scrambled shRNA transfected Hec50co cells, stable MTDH knockdown modestly increased the sensitivity of Hec50co cells to single agent therapy (either LBH589 or TRAIL alone, Figure 4A, B). However, as shown in Figure 4B, a more dramatic induction of sensitivity occurred in stable MTDH knockdown Hec50co cells with the combination of LBH589 and TRAIL. These data suggest that MTDH expression is an important factor underlying resistance to the combined regimen.

### Apoptosis regulators involved in MTDH-mediated cell death

**Figure 5:**
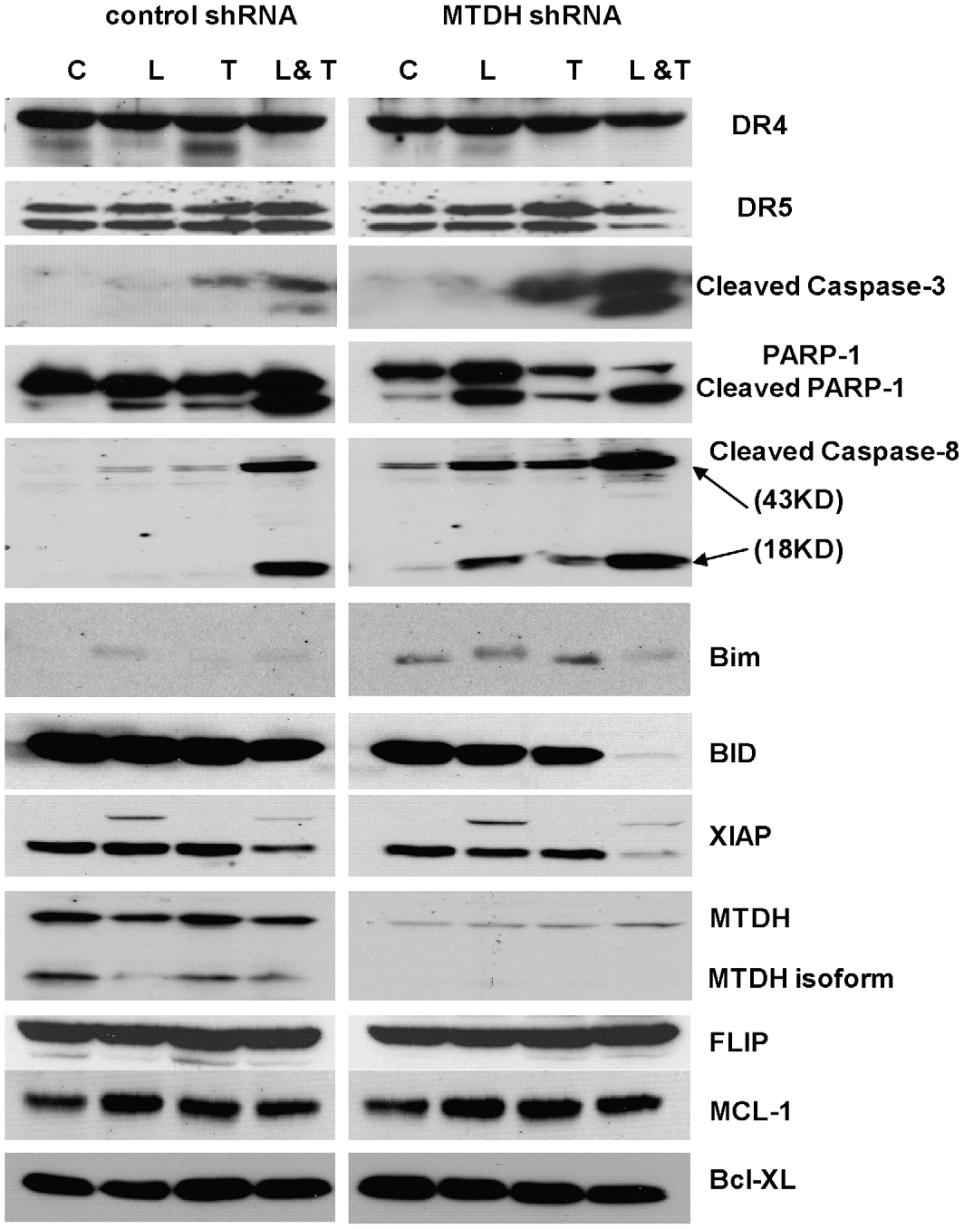
Expression of pro-/anti-apoptosis genes. Control or MTDH knockdown Hec50co cells were treated for 24 hours with vehicle control, 20 nM LBH589, 25 ng/ml TRAIL or LBH589 and TRAIL at the concentrations noted. Lysates were collected. Expression of DR4, DR5, and apoptosis related caspase-3, caspase-8, PARP-1, BID, FLIP, XIAP, Bim, MCL-1 and BCL-XL was analyzed by Western blotting.

To study the molecular mechanism of cell death in MTDH knockdown cells, expression of proteins associated with apoptosis (both pro- and anti-apoptotic factors) were tested in MTDH control and knockdown cells after single treatment (LBH589 or TRAIL) or after combined treatment (LBH589 and TRAIL). The combination treatment did not enhance expression of the TRAIL receptors DR4 and DR5. Additionally, there were no changes in expression of anti-apoptotic genes Bcl-xL, Mcl-1, or FLIP in MTDH control and knockdown Hec50co cells after the combinatorial treatment (Figure 5).

Interestingly, the pro-apoptotic BH3-only protein Bim was up-regulated in untreated MTDH knockdown Hec50co cells compared to control cells (Figure 5), which indicates that depletion of MTDH alone may lead to induction of pro-apoptotic factors. When control Hec50co cells were treated with LBH589 alone or in combination with TRAIL, expression of Bim increased. In contrast, expression of Bim was significantly enhanced in MTDH knockdown Hec50co cells without any treatment, while treatment with either LBH589 or TRAIL further enhanced the expression of Bim. However, Bim expression was slightly decreased in MTDH knockdown cells after co-treatment with LBH589 and TRAIL.

In control cells, post-translational modification of XIAP occurred upon treatment with LBH589, as demonstrated by a higher migrating species, and reduction of both total and modified XIAP was only detected with LBH589 and TRAIL combination treatment (Figure 5). This effect was accentuated when MTDH was knocked down, such that we detected a greater reduction in XIAP post-translational modification in MTDH knockdown Hec50co cells compared to control cells after LBH589 and TRAIL treatment. Furthermore, significant induction of cleavage of caspase-8, caspase-3 and PARP-1 was detected after single treatment with LBH589 or TRAIL, or the combination in MTDH knockdown Hec50co cells compared with control cells. Reduction of Bid was also observed in MTDH knockdown cells treated with the combination of LBH589 and TRAIL. Collectively, these data demonstrate that knockdown of MTDH can increase extrinsic apoptosis induced by TRAIL and LBH589, and the principal mechanism is via up-regulation of Bim. Down-regulation of XIAP and subsequent activation of caspase-8 and caspase-3 as well as downstream substrates such as PARP-1 also occurs.

### Cell cycle checkpoint regulation by MTDH

**Figure 6:**
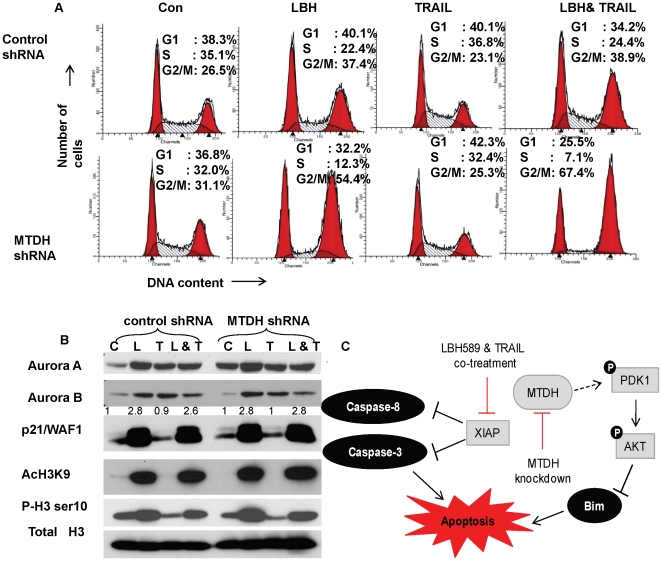
Cell cycle accumulation at G2/M in MTDH knockdown Hec50co cells at 24 hours after LBH589 or LBH589 and TRAIL treatment. (A) Cell cycle profiles were determined in control or MTDH knockdown Hec50co cells at 24 hours after treatment with 20 nM LBH589, 25 ng/ml TRAIL, or LBH589 and TRAIL in combination. The percentage of cells in the G1, S and G2/M phases were determined. (B) The change in Aurora A, Aurora B, p21, histone H3 acetylation at lysine 9, histone H3 phosphorylation at serine 10 and total histone H3 was assessed by Western blotting following 24 hours of treatment with the indicated reagents. Total histone H3 was used as a loading control. (C) A model for the elucidated mechanism by which knockdown of MTDH increases cell death by TRAIL and the HDAC inhibitor LBH589. MTDH knockdown decreases phosphorylation of PDK1 and its substrate AKT, which results in increased expression of the proapoptotic protein Bim. XIAP expression is downregulated in response to combined LBH589 and TRAIL treatment, thereby releasing the XIAP mediated inhibition of caspase-3 and -8 activation. Collectively, the regulation of these pathways promotes apoptotic cell death.

In order to determine the precise mechanism by which MTDH confers resistance to TRAIL and LBH589 treatment, we next investigated the role of MTDH in cell cycle control. Control and MTDH knockdown Hec50co cells that were untreated or treated with TRAIL alone had similar cell cycle profiles (Figure 6A). In contrast, treatment with the HDAC inhibitor LBH589 in cells lacking MTDH resulted in a greater proportion of cells persistently in the G2/M phase. When Hec50co MTDH knockdown cells were treated with both LBH589 and TRAIL, a decreased proportion of cells were in S phase and an increased proportion of cells were arrested in G2/M (Fig. 6A). These data show that MTDH has a significant role in regulating the cell cycle checkpoint in the context of combinatorial TRAIL and HDAC inhibitor treatment.

To investigate the mechanism by which MTDH regulates the cell cycle, expression of cell cycle regulators was measured. Increase of Aurora A at the protein level was observed in MTDH knockdown cells as compared to control cells (Figure 6B). When control and MTDH knockdown Hec50co cells were exposed to LBH589, there was an increase in Aurora A, Aurora B, acetylation of histone H3 at lysine 9, phosphorylation of histone H3 at serine 10, and p53-independent up-regulation of p21 WAF1, the inhibitor of cyclin-dependent kinases (Figure 6B). Interestingly, TRAIL treatment resulted in a substantial decrease in histone H3 phosphorylation, which corresponded to a decrease in the percentage of cells in G2/M phase as compared to untreated cells. Overall these data implicate a novel role for MTDH in regulating cell cycle checkpoints.

## Discussion

Although MTDH has been linked to both metastasis and resistance to chemotherapy in various types of cancers [1], [25], the mechanism and biological function of MTDH is largely unknown. Depletion of MTDH can enhance the effects of chemotherapeutic drugs such as doxorubicin, paclitaxel and 5-FU [10], [11]. Here we report that expression of MTDH is highly elevated in endometrial cancers tissues compared to normal endometrium. Our data demonstrate that depletion of MTDH with a specific shRNA can also increase cellular sensitivity to targeted therapies, including the HDAC inhibitor LBH589, TRAIL and a combination regimen with both agents. As depicted in Figure 6C, we have identified the potential mechanism by which knockdown of MTDH increases cell death in response to treatment with TRAIL and the HDAC inhibitor LBH589. Specifically, cells lacking MTDH have decreased phosphorylation of PDK1 and its substrate AKT, which in turn stimulates expression of the proapoptotic protein Bim. Combined LBH589 and TRAIL treatment results in downregulation of XIAP, thereby allowing activation of caspase-3 and -8. These molecular changes result in apoptotic cell death in cells lacking MTDH. Our data suggest therefore that MTDH-mediated resistance occurs through activation of pro-survival signaling pathways and potentially by aberrant regulation of cell cycle checkpoints.

Using a microarray approach, we identified many candidate genes that are differentially regulated in cells lacking (MTDH shRNA) or expressing (control shRNA) MTDH. Of particular interest were calbindin 1(CALB1) and galectin-1, two proteins that have previously been linked to pro-survival signaling pathways. CALB1 is a calcium binding protein with four active calcium binding domains. CALB1 was reported to interact with Myo-inositol monophosphatase (IMPA) using phage display and to induce IMPA activity up to 250-fold [26], [27]. IMPA is an enzyme that dephosphorylates myo-inositol monophosphate, myo-inositol-1,3-diphosphate, and myo-inositol-1,4-diphosphate to generate free myo-inositol which plays important role in programmed cell death [28]. Galectin-1, a beta-galactosidase binding protein, is strongly expressed in a variety of human cancers and has been shown to play a role in PI3K signaling and Akt activation [29]. In glioblastoma cells, knockdown of galectin-1 impairs the ability of insulin-like growth factor (IGF1) stimulation to elevate cellular PIP3 levels (Perry et al.,2010, AACR 101^st^ annual meeting abstract 301). Our data indicate that MTDH-mediated up-regulation of galectin-1 and CALB1 may be the mechanism by which MTDH stimulates an increase in PIP3 levels, thereby activating the PI3K/AKT/mTOR pathway. This pathway plays an important role in endometrial carcinogenesis, either by the mutation of PTEN or by other mechanisms, constitutive activation is the rule. Our identification of the over-expression of MTDH leading to increased PIP3 levels provides yet another potential explanation for the high activity of PI3K and AKT frequently found in endometrial cancers.

Inhibition of MTDH was shown to increase cell death induced by LBH589 and TRAIL combination treatment (Figure 4). TRAIL can induce extrinsic apoptosis via directly binding with death receptors. However, while TRAIL can selectively induce apoptosis in endometrial cancer cells, it cannot induce apoptosis in normal endometrial tissues at the same concentration. Furthermore, the concentration of TRAIL (200 ng/ml) necessary to induce apoptosis in endometrial cancer cells is much higher than that previously used for cells from other solid tumors (e.g. 25–50 ng/ml in pancreatic cancer) [13], [30]. This indicates potential baseline resistance to TRAIL in endometrial cancer which could be linked to high MTDH expression. To overcome resistance, several chemotherapeutic agents or targeted inhibitors that have been reported to increase sensitivity to TRAIL-induced apoptosis have been used in combinatorial regimens [12]. Here we demonstrate that the HDAC inhibitor LBH589 can increase TRAIL-mediated caspase-8 activation and subsequent extrinsic cell death in endometrial cancer cells. Knockdown of MTDH can further stimulate caspase-8 cleavage induced by LBH589, TRAIL, or the combination of the two. An increase in XIAP post-translation modification, a reduction in total XIAP, and induction of the pro-apoptosis gene Bim were observed in cells treated with the LBH589 alone. Loss of Bid was observed in MTDH knockdown cells after LBH589 and TRAIL combination treatment, which is likely due to Bid activation via cleavage. The finding of decreased Bim with the same treatment was somewhat unexpected. However, our data in Figure 3 suggest that MTDH knockdown negatively regulates the Akt pathway, which may initially result in elevated expression of Bim. As cells progress through apoptosis, less stable proteins may be subject to degradation. Since MTDH knockdown cells have accelerated cell death in response to the combination treatment (Figure 4), it is possible that the decrease in Bim is a reflection of rapid protein turnover.

In response to LBH589 treatment alone or with TRAIL, the two principal effects of MTDH knockdown on cell cycle were a decrease in the percentage of cells in S and a concomitant increase in the percent of cells in G2/M, where the cells appear to be arrested. At the cellular level, however, knockdown MTDH alone in the absence of therapy did not affect the expression of canonical cell cycle regulatory proteins. For example, LBH589 significantly enhanced histone H3 acetylation on lysine 9 (Figure 6B) which may permit the transcriptional activation of the p21 gene [31]. However, H3 modification was not affected by shRNA knockdown of MTDH. Proteasome-mediated degradation of Aurora A, Aurora B and c-FLIP was reported to be responsible for the increase in sensitivity to TRAIL by HDAC inhibitor treatment in some cancer cells [15], [32]. However, no significant changes in Aurora A and B or c-FLIP levels were observed in endometrial cancer cells treated with LBH589 in our studies.

This is the first report of MTDH's impact on resistance in targeted therapy for endometrial cancer. For these studies, we chose the combination of LBH589 and TRAIL because of the impact of these agents on proapoptotic pathways. We demonstrate herein that knockdown of MTDH sensitizes cells to programmed cell death though a mechanism that involves inhibition of PDK1 and Akt phosphorylation, increase in Bim, and reduction in XIAP. Therefore, we can target MTDH to enhance sensitivity to not only chemotherapy but also targeted agents that work through programmed cell death. These data provide compelling evidence that, by downregulating MTDH in endometrial cancer, we can magnify the proapoptotic pathways and response to therapy.

## Materials and Methods

### Ethics Statement

Normal endometrial tissues and endometrial tissues from endometrial cancers were collected with the consent of patients under the University of Iowa Institutional Review Boards approved protocol. We obtained informed written consent from all participants involved in this study.

### Cell Lines and Cell Culture

Human endometrial cancer cell lines Hec50co, RL95, AN3CA, ECC1, Ishikawa H, and KLE were maintained as described previously [33]. Cells were cultured in DMEM medium containing 10% fetal bovine serum and 1% penicillin/streptomycin (all from Gibco BRL, Carlsbad, CA) at 37°C in a humidified atmosphere of 5% CO_2_ and 95% air. To generate an MTDH knockdown model, Hec50co cells were transfected with MTDH sh RNA or control non-silencing shRNA constructs per the manufacturer's instructions (ORIGENE, Rockville, MD). Individual cell clones resistant to puromycin were expanded and subjected to screening for MTDH expression by Western blotting.

### Endometrial Tissues and Tissue Arrays

Normal endometrial tissues and endometrial tissues from endometrial cancers were collected from the University of Iowa Hospitals and Clinics. The endometrial disease spectrum (endometrial cancer progression) tissue array (UT 801) was purchased from US Biomax, Inc. (Rockville, MD).

### Reagents, Antibodies and Western blotting

Human recombinant TRAIL (TNF-related Apoptosis inducing ligand) was purchased from R&D Systems, Inc. (Minneapolis, MN). LBH589 was purchased from Selleck (Houston, TX). The following antibodies were used: anti-MTDH and anti-β-actin antibodies were from Sigma (St Louis, MO, USA); and anti-cleaved caspase-3, caspase-8, DR4, DR5, Bim, Bid, FLIP, XIAP, p21, PARP-1 p-AKT S473, p-AKT T308, PDK1, p-PDK1 S204, histone 3, p-histone 3 serine10 and histone 3 lysine 9 acetylation antibodies were from Cell Signaling Technology Inc. (Danvers, MA). Whole-cell protein lysates were prepared and analyzed by Western blotting as previously described [34].

### Expression Array and RT-qPCR Analysis

Total RNA was isolated from MTDH knockdown and control endometrial cancer cell lines (in biological triplicates for control and MTDH knockdown cells) using the *mir*Vana miRNA Isolation Kit (Ambion, Austin, TX), and cDNA was generated using the High-Capacity cDNA Reverse Transcription Kit (Applied Biosystems, Carlsbad, CA). A human gene array 1.0 ST (Affymetrix, Santa Clara, CA) was interrogated using the biological triplicates in the DNA Core Facility at the University of Iowa, and Partek Genomics Suite (Partek Inc, St. Louis, MO) software was used to generate gene expression values. All data is MIAME compliant and the raw data has been deposited in a MIAME complaint database GEO, as detail on the website http://www.ncbi.nlm.nih.gov/projects/geo/query/acc.cgi?acc=GSE26134. The accession number for Hec50co cells is GSE26134 and for Ishikawa H cells is GSE27828. Several MTDH downstream genes were randomly chosen to validate the microarray data using TaqMan primers and probes (Applied Biosystems, Carlsbad, CA) for quantitative RT-PCR analysis. Experiments were performed using biological triplicates.

### Cell Survival Assays

Cells were seeded in 96-well cell culture plates and treated the next day with the agents as indicated. The number of viable cells was determined using the WST-1 assay (Clontech Laboratories Inc.), as previously described [35].

### Cell Cycle Analysis

DNA content analysis by flow cytometry has been described previously [36], [37].

## Supporting Information

Table S1 **mRNAs that are more than two fold increased in control shRNA expressed cells compared with MTDH shRNA expressed Hec50co cells.**
(TIF)

Table S2 **mRNAs that are more than two fold decreased in control shRNA expressed cells compared with MTDH shRNA expressed Hec50co cells.**
(TIF)

Table S3 **mRNAs that are more than five fold increased in control shRNA expressed cells compared with MTDH shRNA expressed Ishikawa H cells.**
(TIF)

Table S4 **mRNAs that are more than five fold decreased in control shRNA expressed cells compared with MTDH shRNA expressed Ishikawa H cells.**
(TIF)
