# Supplementary figures and images for: Knockdown of MTDH Sensitizes Endometrial Cancer Cells to Cell Death Induction by Death Receptor Ligand TRAIL and HDAC Inhibitor LBH589 Co-Treatment
Source: PLoS One. 2011 Jun 8;6(6):e20920. doi: 10.1371/journal.pone.0020920 (PMC3110819; doi:10.1371/journal.pone.0020920)

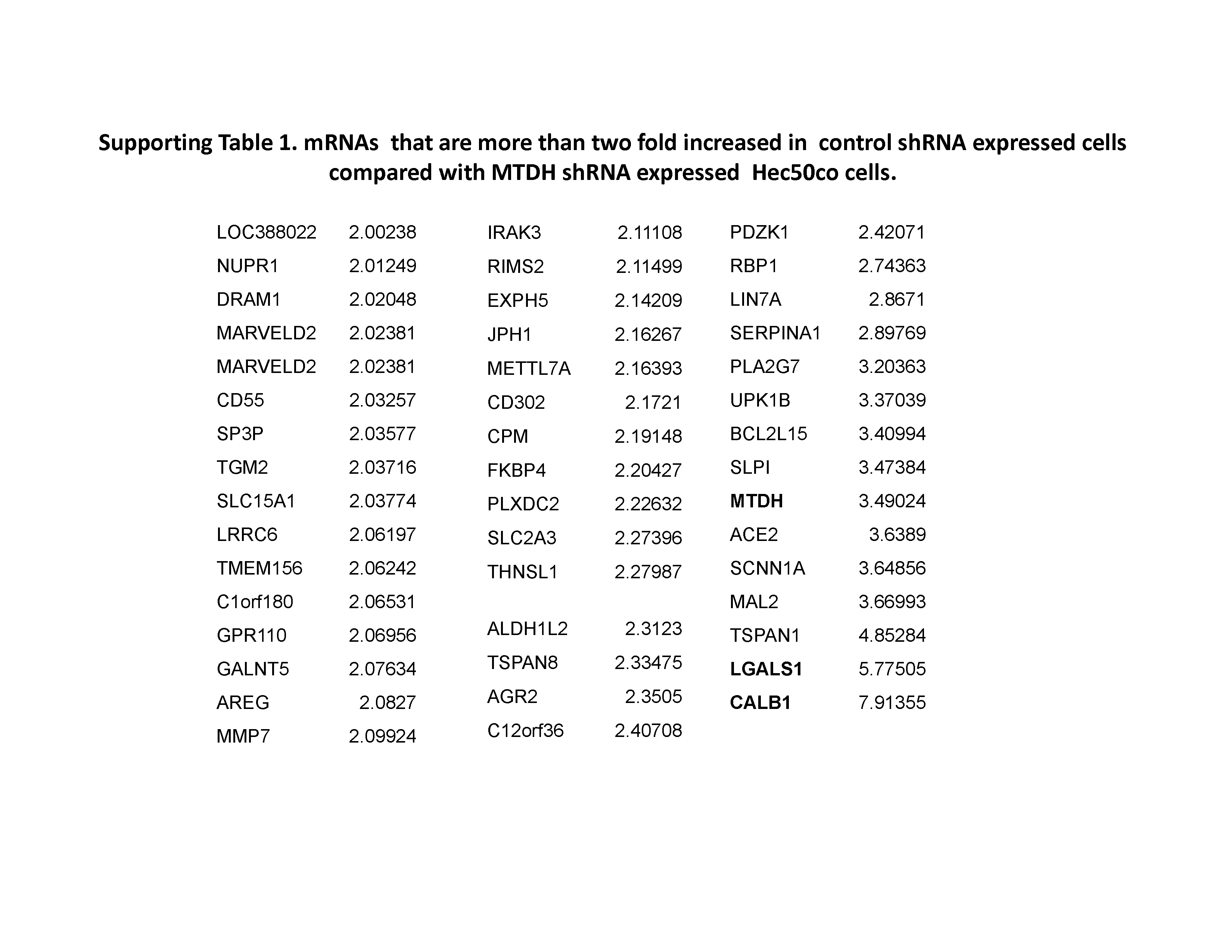

Supplement: Table S1 — mRNAs that are more than two fold increased in control shRNA expressed cells compared with MTDH shRNA expressed Hec50co cells. (TIF) [file pone.0020920.s001.tif]

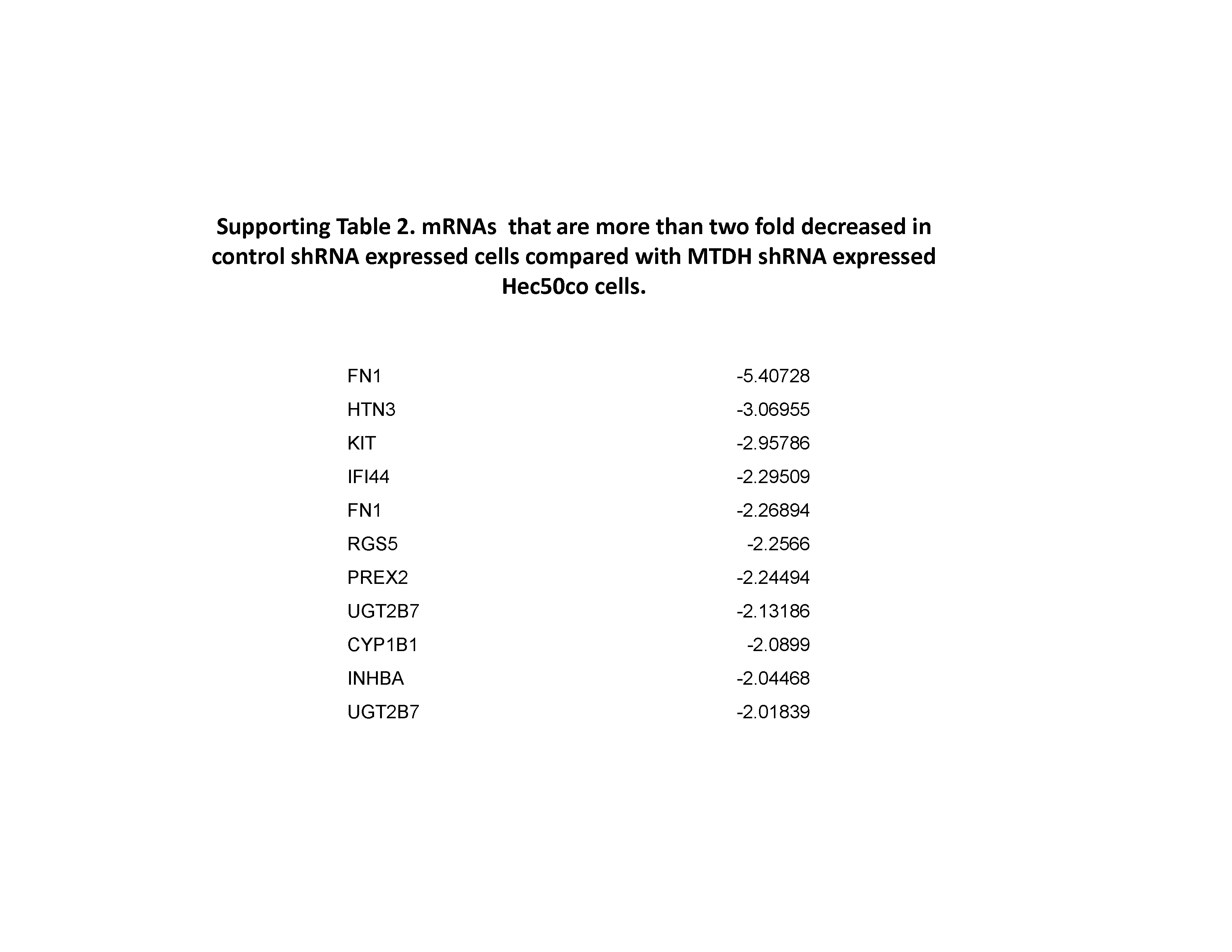

Supplement: Table S2 — mRNAs that are more than two fold decreased in control shRNA expressed cells compared with MTDH shRNA expressed Hec50co cells. (TIF) [file pone.0020920.s002.tif]

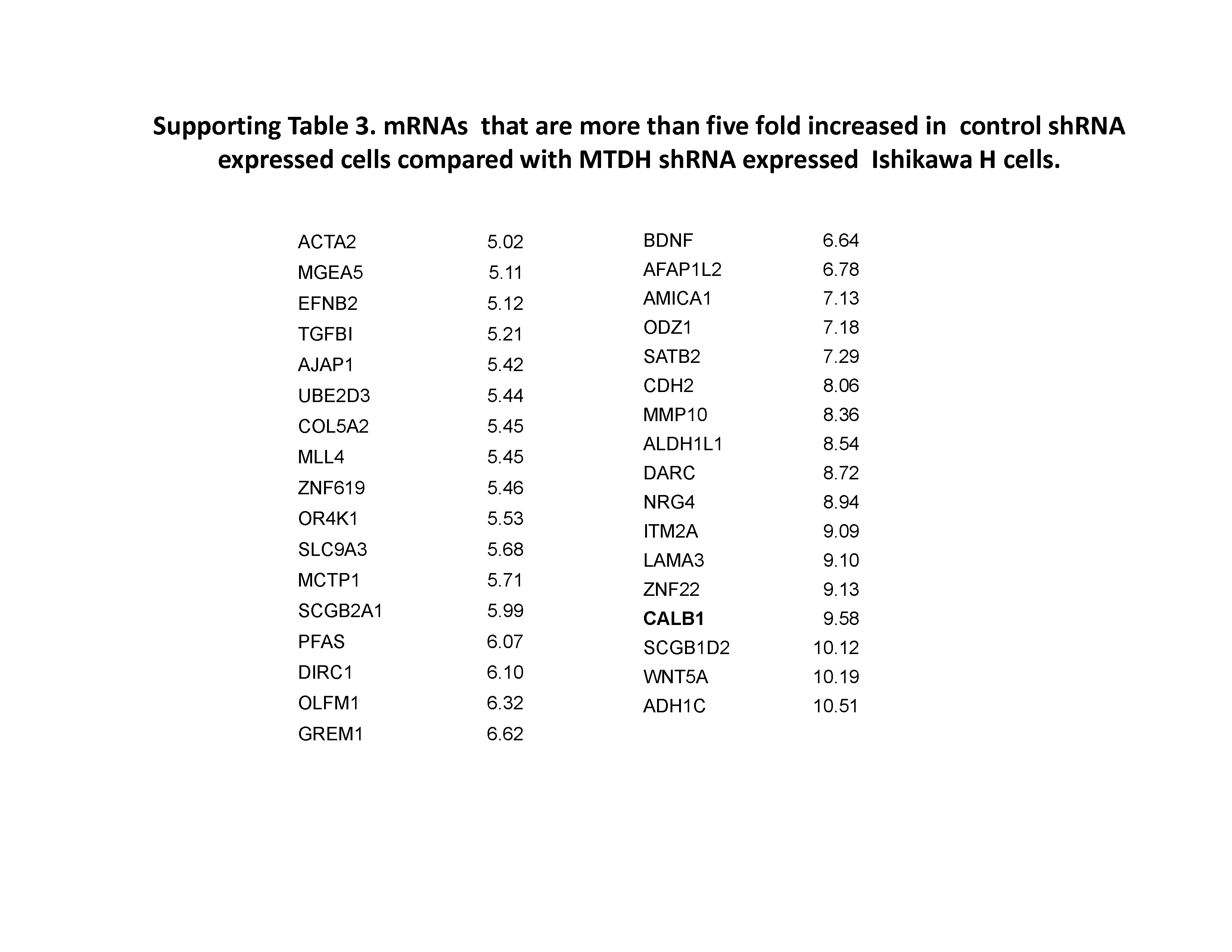

Supplement: Table S3 — mRNAs that are more than five fold increased in control shRNA expressed cells compared with MTDH shRNA expressed Ishikawa H cells. (TIF) [file pone.0020920.s003.tif]

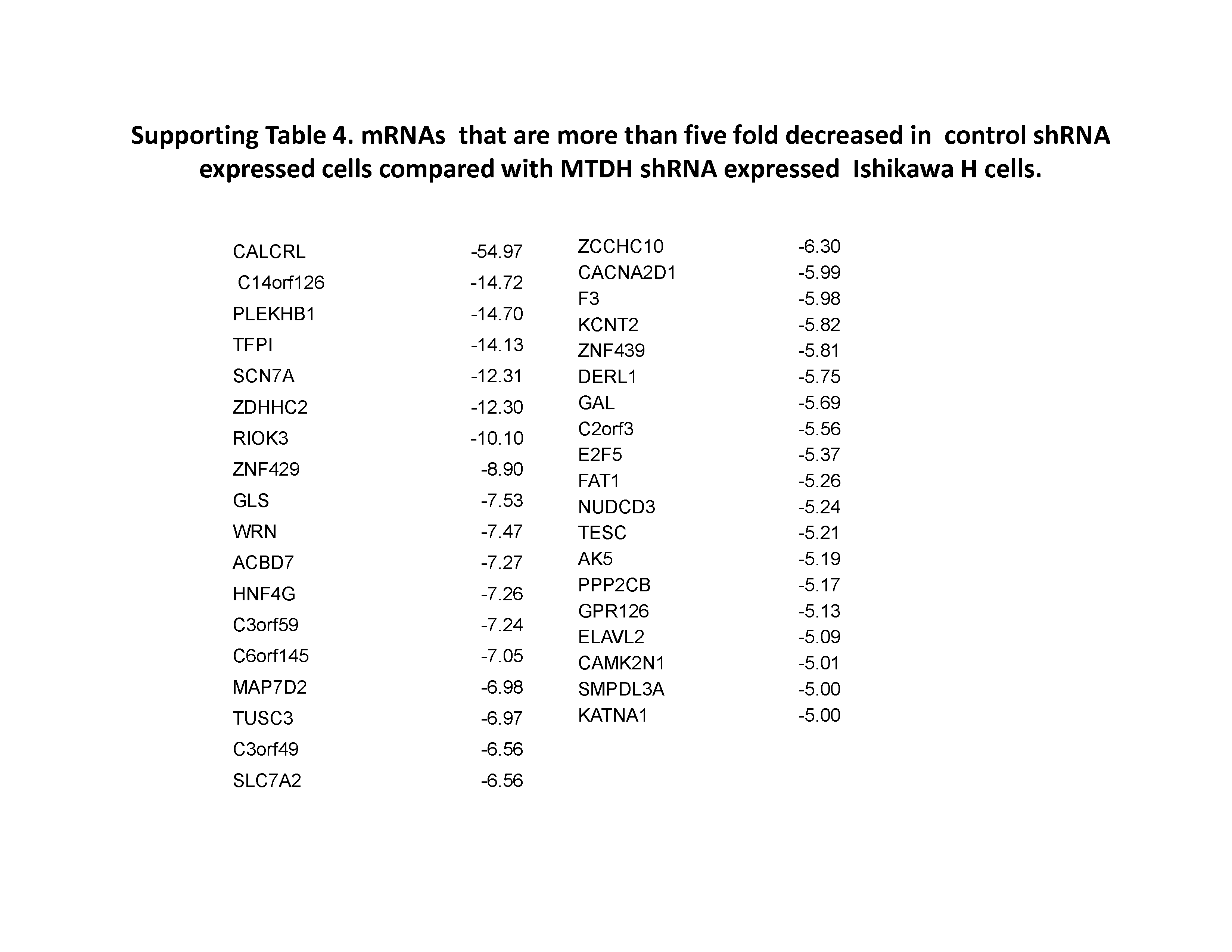

Supplement: Table S4 — mRNAs that are more than five fold decreased in control shRNA expressed cells compared with MTDH shRNA expressed Ishikawa H cells. (TIF) [file pone.0020920.s004.tif]
